# Polypropylene/ZnO Nanocomposites: Mechanical Properties, Photocatalytic Dye Degradation, and Antibacterial Property

**DOI:** 10.3390/ma13040914

**Published:** 2020-02-19

**Authors:** Ampawan Prasert, Somchoke Sontikaew, Dilok Sriprapai, Surawut Chuangchote

**Affiliations:** 1 Department of Tool and Materials Engineering, Faculty of Engineering, King Mongkut’s University of Technology Thonburi, 126 Prachauthit Rd., Bangmod, Thungkru, Bangkok 10140, Thailand; ampawan.p@mail.kmutt.ac.th (A.P.); somchoke.son@kmutt.ac.th (S.S.); dilok.sri@kmutt.ac.th (D.S.); 2 Research Center of Advanced Materials for Energy and Environmental Technology, King Mongkut’s University of Technology Thonburi, 126 Prachauthit Rd., Bangmod, Thungkru, Bangkok 10140, Thailand

**Keywords:** nanocomposite, polypropylene, zinc oxide, photocatalysis, antibacterial

## Abstract

Nanocomposite materials were prepared by compounding polypropylene (PP) with zinc oxide (ZnO) nanoparticles, using a twin-screw extruder. The compound was molded by injection molding to form dumbbell-shaped specimens. The influence of ZnO nanoparticle content on the morphology, mechanical properties, chemical structure, photocatalytic activity, and antibacterial properties of the obtained nanocomposites was investigated. The morphological images showed that the ZnO nanoparticles were well distributed in the PP matrix. Characterizations of the mechanical properties and chemical structures before and after sunlight exposure found that at the shortest exposure time, crosslinks could occur in the nanocomposites, which resulted in improved mechanical properties. However, sunlight exposure with the time period longer than 18 weeks caused the reduction of the mechanical properties, due to degradation of the PP matrix. It was found that PP with 2% ZnO could achieve the photocatalytic degradation of methylene blue up to 59%. Moreover, the result of antibacterial tests indicated that the nanocomposites had better antibacterial properties than neat PP.

## 1. Introduction

In the midst of the progress of science and technology, the interest in materials science has been rapidly increasing in accordance with human needs and environmental concerns. Simultaneously, environmental responsibility is necessitating that consumers find solutions to avoid the accumulation of materials as waste.

Presently, composite materials that consist of materials with at least two different properties have been widely used, due to the fact that the properties of the composites are better than those of the original components individually [1,2]. One type of composite materials is polymer nanocomposites with combination of a nanostructured photocatalyst (as a filler) and a polymer matrix. Photocatalysts can participate in photocatalytic reactions to form oxidative species, i.e. superoxide radical (O_2_^−^), hydrogen peroxide (H_2_O_2_) and hydroxyl radical (HO), by UV light, which are extremely reactive for photodegradation processes [3]. Photocatalyst-filled nanocomposite materials show enhancement of the physical and mechanical properties of the matrix and exhibit excellent photocatalytic activity as photocatalysts due to the distribution of the catalysts [2]. With various factors, these materials have been often utilized in various industries, especially food packaging [4,5], medical devices [6,7] and water transport pipes [8].

Recently, several studies on nanocomposite materials with polymer matrixes, such as polyethylene (PE) [9,10], polypropylene (PP) [11,12,13], polylactic acid (PLA) [14], and polyvinyl alcohol (PVA) [15,16] have been reported. One commercial thermoplastic polymer that has been widely used in various industries is polypropylene (PP), because of its low density, low cost, easiness in process, chemical resistance, and also excellent mechanical properties [12,17]. With the great properties of PP, it has been frequently combined with nanostructured photocatalysts, i.e., ZnO [2,18,19] and TiO_2_ [11,20], to fabricate nanocomposites. ZnO nanoparticles are promising photocatalysts with high photocatalytic activity, biocompatibility, and nontoxicity to human cells [21,22,23], so they have been extensively applied in the diverse areas [3,12,16,24,25] such as sunscreens, paints, and coatings in energy and environmental applications [26]. ZnO nanoparticles also show strong antibacterial properties (in the mixtures with organic compounds [3,27]) and high specific surface area to volume ratios [28].

With the focus on environmental issues, the use of plastics and their degradation pathways are of concern. Polymer composites with suitable mechanical properties during use and with a shorter degradation time are under development. Some studies on the degradation of PP in the presence of ZnO under stimulated UV-light irradiation have been reported [2]. However, the degradation of PP with ZnO after exposure to actual sunlight in an outdoor environment has not been studied. The mechanism of UV-induced degradation of PP driven by ZnO nanoparticles has been studied and proposed [2]. The mechanical properties of the composites after the actual exposure to sunlight and the relations with chemical structure changes and the degradation mechanism, however, have not been demonstrated. In this study, we prepared dumbbell-shaped PP/ZnO to demonstrate the molded-products of the photocatalyst/polymer nanocomposites. Different from other reports, a compound of PP/ZnO was formed by a twin-screw extruder and it was injected into a mold with a dumbbell-shaped cavity by injection molding. The influence of ZnO nanoparticles on the morphology, mechanical properties, and changes in chemical structures after exposure to sunlight have been investigated. The photocatalytic activity and antibacterial properties of the nanocomposite have also been studied.

## 2. Materials and Methods

### 2.1. Materials

Polypropylene (PP) was purchased from IRPC Public Company Limited (Bangkok, Thailand) under the trade name of POLYMAXX^®^ (3342M). Zinc oxide (ZnO) nanoparticles (average particle size = 72 ± 18 nm, purity = 99.5%) were supplied by Nano Materials Technology Limited, (Chonburi, Thailand). Methylene blue (MB) (Sigma-Aldrich, St. Louis, MO, USA) solution was prepared to the desired concentration in deionized (DI) water.

### 2.2. Methods

#### 2.2.1. Preparation of PP/ZnO Nanocomposite Materials

Before mixing, PP pallets and ZnO were dried in an oven (80 °C, 12 h) to get rid of moisture then cooled down to room temperature. PP pellets and ZnO nanoparticles were mixed by a high-speed mixer for the homogeneity of samples and then compounded by a twin screw extruder (polylab CTW Haake, Karlsruhe, Germany). The temperatures of the extruder from the hopper to the die were 175, 180, 185, and 190 °C. The contents of ZnO nanoparticles in the composites are varied as 0.5, 1, and 2 wt.%. For the comparative aim, the samples of PP/0%ZnO (neat PP) were also subjected to the same processing. The PP and composite compounds were then injected into a dumbbell-shaped mold with dimensions of 12 × 163 × 3 mm by an injection molding machine (ES 200/50HL, Engel, Stuttgart, Germany). From now on, the composites with the contents of 0, 0.5, 1, 2 wt.% of ZnO nanoparticles are designated as PP/0%ZnO, PP/0.5%ZnO, PP/1%ZnO, PP/2%ZnO, respectively.

#### 2.2.2. Characterizations of PP/ZnO Nanocomposite Materials

1. Scanning Electron Microscopy (SEM)

The nanoparticles of ZnO and morphological appearances of PP/ZnO nanocomposites were observed using a scanning electron microscope (JSM-6610 LV, JEOL, Tokyo, Japan). The nanocomposites were immersed in liquid nitrogen and cracked for cross-sectional observation. Before the observation, the ZnO particles and nanocomposite samples were coated with Au using a sputter coater (108 Auto, Cressington, Watford, UK) for the high solution images. Energy dispersive x-ray spectroscopy (EDS, INCA-xart, Oxford, Abingdon, UK) was used for the ZnO elemental analysis.

2. Mechanical Property Test

Dumbbell-shaped specimens prepared by the injection molding were evaluated by tensile testing according to ASTM D638 method at room temperature with a crosshead speed 25 °C and 100 mm/min, respectively. A universal testing machine (UTM, LR 50K, LLOYD, Bognor Regis, UK) was used to obtain the Young‘s modulus, stress at yield point, and elongation at break point of PP/ZnO nanocomposites. Note that in the case of some specimens that the stress at yield point of nanocomposites could not be obtained (the specimens broke before the yield point), the maximum stress (at break) was reported instead.

The specimens were continuously subjected to sunlight (outdoor environment) for 24 weeks from September 2018 to March 2019 in Bangkok, Thailand. During the exposure time, the UV index and maximum-minimum temperatures were 8–14 and 22–39 °C, respectively [29]. During that period of time, the specimens were taken at 3, 6, 12, 18, and 24 weeks for the tensile tests.

3. Fourier-Transform Infrared Spectroscopy (FTIR)

Molecular changes in PP/ZnO composite samples upon exposure to sunlight were investigated using a 6700 FTIR instrument (Thermo Nicolet, Waltham, Massachusetts, USA) in attenuated total reflectance (ATR) mode in the range of wavenumbers between 4000 and 400 cm^−1^. The relative changes in C–C stretching (1166 cm^−1^), carbonyl species (1800–1600 cm^−1^), C–H stretching (2800–2900 cm^−1^), and hydroperoxide (3600–3200 cm^−1^) were monitored by the calculations of the chemical structure index using the peak at 1377 cm^−1^ as a reference according to Equation (1) [2]:

Chemical structure index = Intensity of the considered peak/Intensity of the reference peak (1377 cm^−1^), (1)

4. Photocatalytic Tests

The photocatalytic test was investigated by the degradation of methylene blue (MB) under UV irradiation. MB is used as a model dye in this work because the mechanism of photocatalytic degradation of MB is quite well known and the color change of MB does not come from complex changes of the MB structure. The reaction in this work was carried out under UVA exposure by UVA lamps with 400 W, maximum wavelength (λ_max_) 365 nm in a wood box. PP/ZnO nanocomposite specimens with dimensions of 1 cm × 1 cm were cut from the dumbbell-shaped samples fabricated by injection molding. Methylene blue solution (25 mg/L) was mixed and stir into a Pyrex cylindrical double-walled reactor with the ratio of the composite to methylene blue solution of 10 g/100 mL. The PP/ZnO nanocomposite suspension was stirred and the temperature of the suspension was controlled throughout this experiment. Before turning on the light, the nanocomposite suspension was stirred under dark conditions to achieve the sorption (desorption and adsorption) equivalence of methylene blue on the photocatalyst surface. 5 mL of nanocomposite suspension sample was taken into a syringe after 30 min in the dark condition. After turning on the light, the nanocomposite suspension sample (5 mL) was taken into a syringe at 30 min and at every 1 h. The concentration (C) of methylene blue was monitored by the absorbance obtained from a spectrophotometer (Spectronic 20 GENESYS, Thermo Electron Corporation, Waltham, MA, USA) at the wavelength of 664 nm [18]. Degradation of methylene blue (%) was determined using Equation (2) [30]:

Degradation of methylene blue (%) = (C_0_ − C_t_)/ C_0_ × 100, (2)

where, C_0_ is an initial concentration of nanocomposite suspension sample after 30 min in the dark condition, and C_t_ is the concentration of nanocomposite suspension sample with the sampling time.

5. Antibacterial Test

The antibacterial activity of PP/ZnO nanocomposite was evaluated using *Escherichia coli*. (*E. coli,* KMUTT Scientific Instrument Center for Standard and Industry, Bangkok, Thailand) as a test microgram. Before the test, the dumbbell-shaped specimens of neat PP (PP/0%ZnO) and PP/2%ZnO nanoparticles were cleaned with alcohol and cut into dimensions of 4.0 cm × 1.2 cm. The test was spilt into two experimental conditions which were dark and daylight conditions for 24 h. The distance between PP/ZnO nanocomposite with *E. coli* and the light source as day-light conditions were set at the same distance of photocatalytic test (25 cm). After 24 h, the microbial resistance was determined by the swab test method [31] and the microbial colonies were counted.

## 3. Results and Discussion

### 3.1. Characterizations of PP/ZnO Nanocomposites

**Figure 1:**
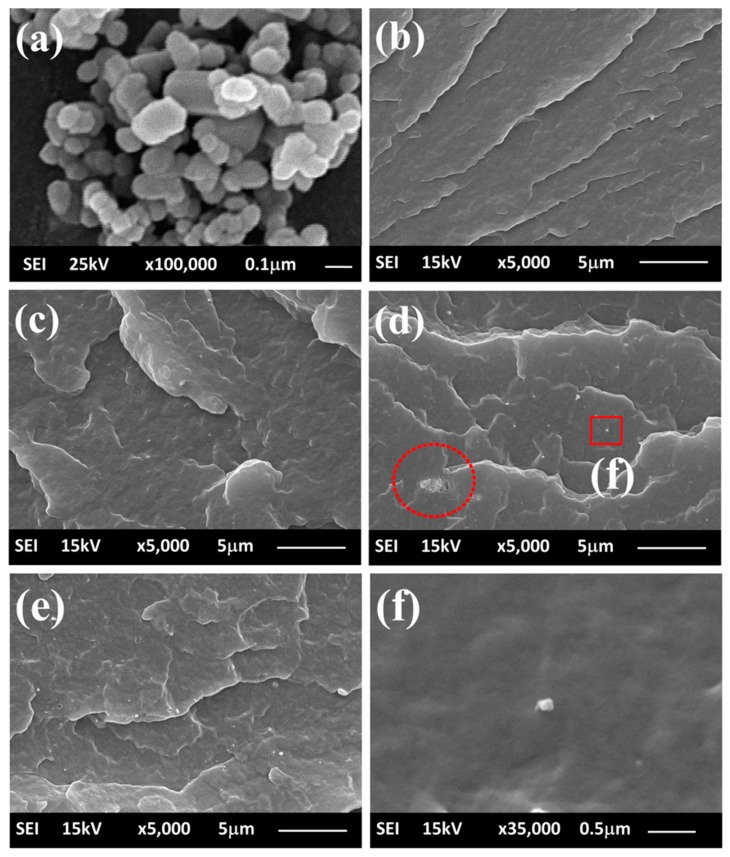
SEM images of (**a**) ZnO nanoparticles and (**b**–**d**) fractured surfaces of PP/ZnO nanocomposite materials: (**b**) PP/0%ZnO, (**c**) PP/0.5%ZnO, (**d**) PP/1%ZnO, and (**e**) PP/2%ZnO [note: (**f**) is a magnified image of the inset of (**d**)].

Figure 1a shows ZnO particles with an average diameter around 72 ± 18 nm. This small size can benefit to increase in specific surface area of the fillers [32].

**Figure 2:**
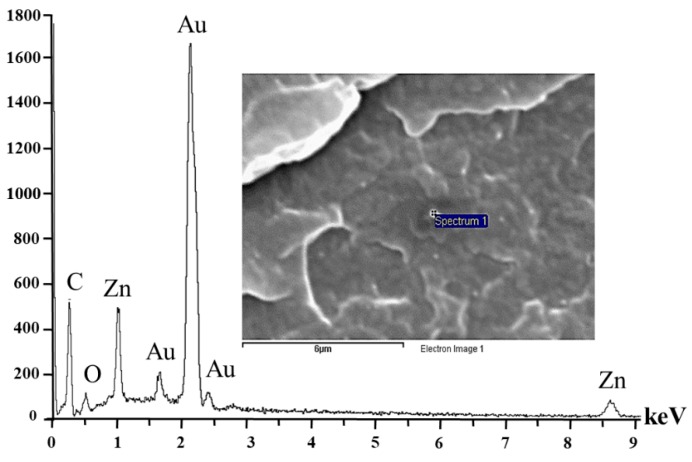
EDS of PP/2%ZnO nanocomposite materials (note: inset is a SEM image of the analyzed point).

Figure 1b,d present the cross-sectional cracked surface of nanocomposite materials that was broken in liquid nitrogen. Particles (white spots) were found in the PP/ZnO nanocomposite samples as magnified in Figure 1f. The number of particles was found to increase with increasing amount of ZnO component (0.5%–2%) in the PP/ZnO nanocomposites. The size of the particles found is less than 100 nm, which is in line with the size of the ZnO used. The EDS result (Figure 2) could confirm that the particles obtained is ZnO. Few agglomerations of nanostructured fillers are generally found in nanocomposites due to the divergence of polarity between the nonpolar matrix and polar fillers, and the small size of fillers also causes high surface energy [4]. Anyway, quite good dispersion of ZnO nanoparticles could be found in the PP/ZnO composites in this work.

### 3.2. Effect of Exposure to Sunlight on Mechanical Property and Chemical Structure Changes

In general, changes in both the mechanical properties and chemical structure can be found in the advancement or degradation of composite materials, caused by many factors such as heating, biological sources, chemical attacks, and sunlight or high energy radiations [17]. The use of ZnO nanoparticles in PP was previously reported in the literature. For example, Jiang et al. [33] investigated UV resistance properties of zeolite/ZnO/iPP composites. The UV influence on PP/ZnO nanocomposite fibers was examined by Karami et al. [34], and Senatova et al. [35] investigated the effect of UV-radiation on structure and properties of PP nanocomposites. The effect of actual sunlight exposure, however, has not been reported yet. Therefore, in this work, it was examined by exposing the PP/ZnO composites to sunlight for about 24 weeks from September 2018 to March 2019 in Bangkok, Thailand. During this test, the weather UV index and maximum and minimum temperatures were 8–14, 22–39 °C, respectively [29].

#### 3.2.1. Tensile Testing Result

**Figure 3:**
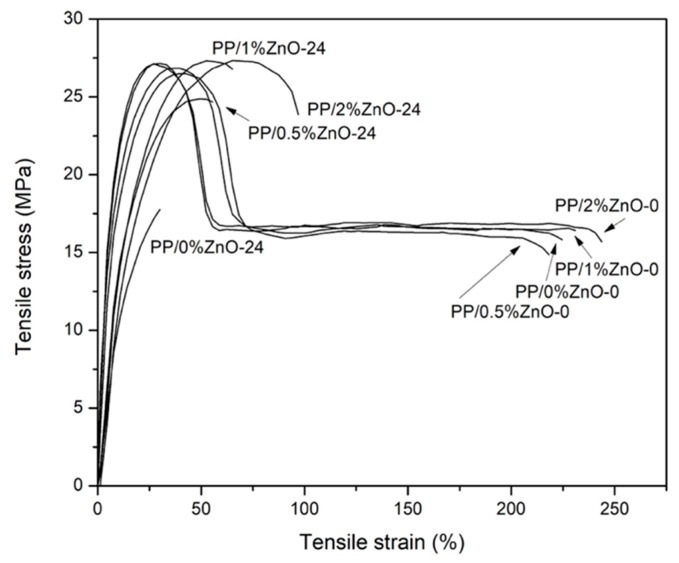
Tensile stress-stain curves of all PP/ZnO nanocomposites exposed to sunlight for 0 and 24 weeks.

Tension force was applied to dumbbell-shaped specimens of PP/ZnO nanocomposites at an appropriate speed until they broke using a UTM. Figure 3 demonstrates the tensile stress-strain curves of as-prepared PP/ZnO nanocomposites compared with the nanocomposites obtained after the exposure to sunlight for 24 weeks. It was found that the overall mechanical properties of PP/ZnO nanocomposites were dramatically reduced after the exposure. The tough polymer behavior with high ductility of the PP nanocomposite changed to more brittle ones after the exposure.

**Figure 4:**
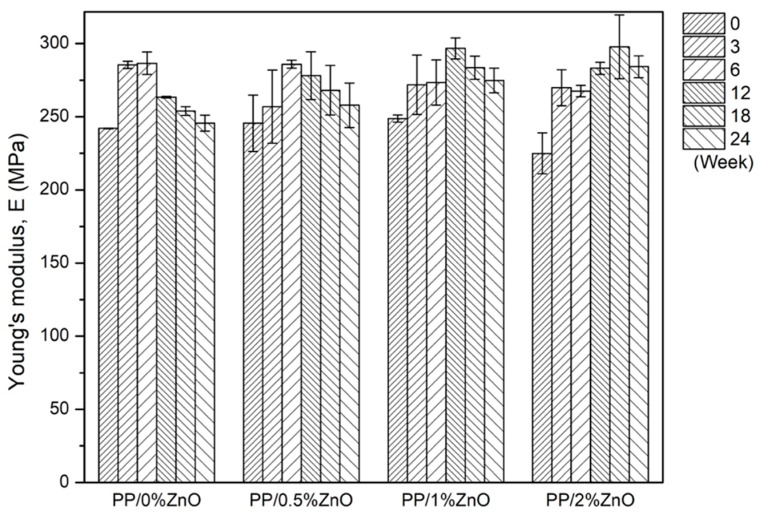
Young’s modulus of PP/ZnO nanocomposite materials at various ZnO contents (from 0%–2%) during exposure to sunlight (note: error bars are standard deviations).

**Figure 5:**
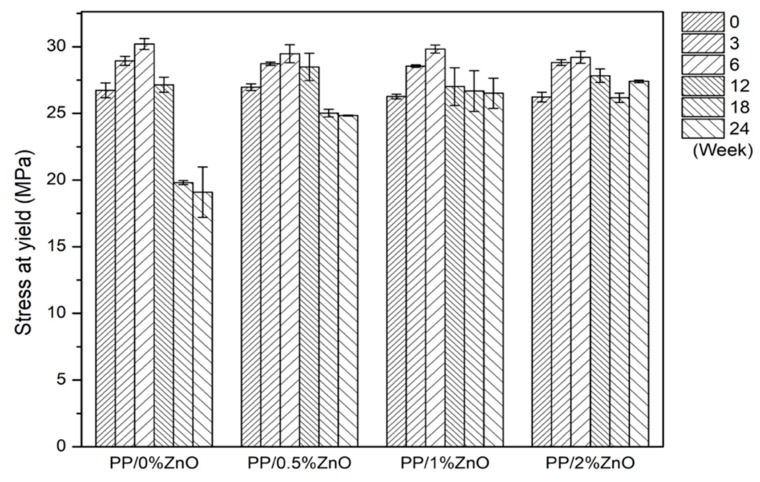
Stresses at yield points of PP/ZnO nanocomposite materials at various ZnO contents (from 0%–2%) during exposure to sunlight (note: (1) error bars are standard deviations; (2) in the case that the specimens broke before the yield point (weeks 18 and 24), the values of maximum stress were reported).

**Figure 6:**
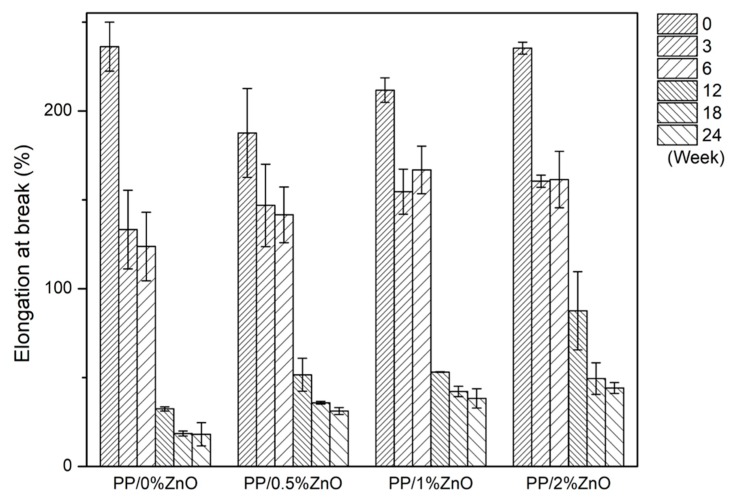
Elongations at break points of PP/ZnO nanocomposite materials at various ZnO contents (from 0%–2%) during exposure to sunlight (note: error bars are standard deviations).

Young’s modulus, stress at yield, and elongation at break were calculated from the tensile testing results obtained. The calculated mechanical characteristics of PP/ZnO nanocomposites after exposure to sunlight for a continuous period from 0 to 24 weeks are shown in Figure 4, Figure 5 and Figure 6. For the as-prepared nanocomposites, Young’s modulus (generally indicates stiffness of materials) increased with increasing ZnO content (consider PP/0.5%ZnO and PP/1%ZnO compared with PP/0%ZnO) due to the increase in filler material.

This is because, at relatively low contents of ZnO with good filler distribution, a high interfacial interaction between ZnO nanoparticles and PP matrix occurred to delay the massive shear yielding along the tensile loading direction [17]. With increasing ZnO to PP/2%ZnO, the modulus was found to decrease, caused by lower effectiveness of the stress transfer between matrix and filler. This could be explained by the weakening effect [34] due to stress concentration of nanoparticles at relatively higher content of ZnO. For the elongation at break, it was found the inverted trend when compared with the modulus because the increase in stiffness reduces the ductility.

The studies of mechanical properties of nanocomposites after exposure to sunlight found that PP/ZnO nanocomposite shows the effect in two ways. After a short exposure time (0–12 weeks) of the PP/ZnO nanocomposite materials, the modulus and yield stress were found to increase, while when the sunlight exposure time was longer than 18 weeks, the modulus and yield stress were found to decrease.

#### 3.2.2. FTIR Results

Changes to C–C bonds, C–H bonds, O–H bonds and carbonyl species can represent the PP degradation by photocatalytic processes that lead to the formation of crosslinking or other low molecular weight compounds, such as carbonyl compounds (alcohol, ketones, carboxylic acids) and hydroperoxide molecules [2], PP/ZnO nanocomposite samples exposed to sunlight for a continuous period were subjected at 3, 6, 12, 18, and 24 weeks to FTIR tests over a wave number range of 4000–400 cm^−1^. The changes in C–C stretching, carbonyl species, C–H stretching, and hydroperoxide bands could were monitored by the FTIR peaks at 1166, 1800–1600, 2800–2900, and 3600–3200 cm^−1^, respectively.

**Figure 7:**
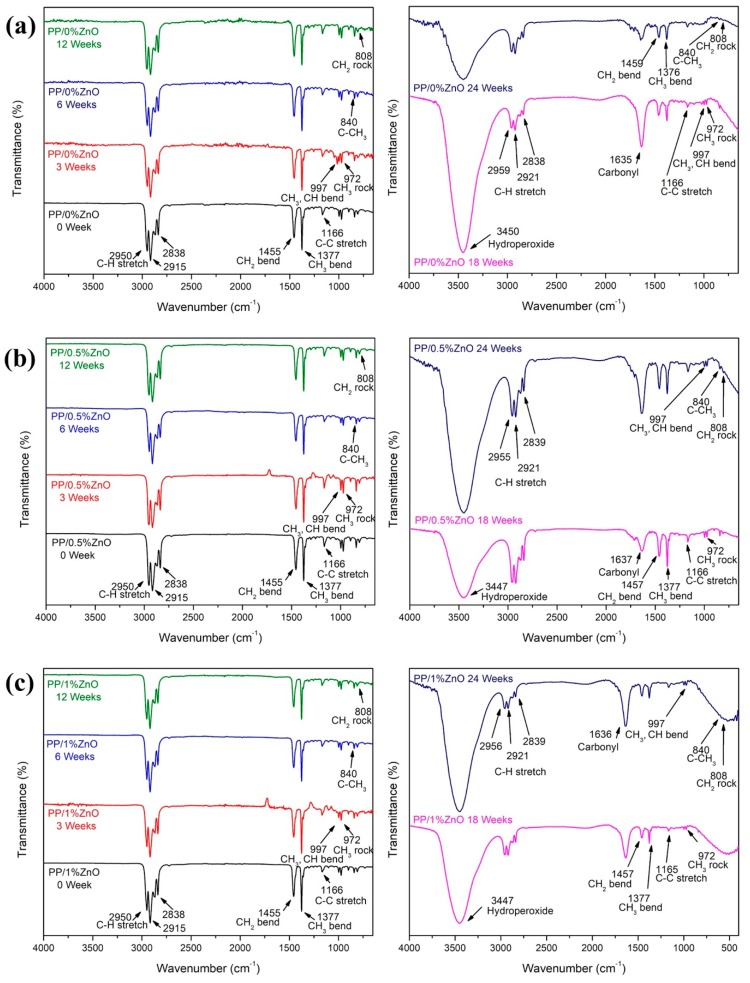

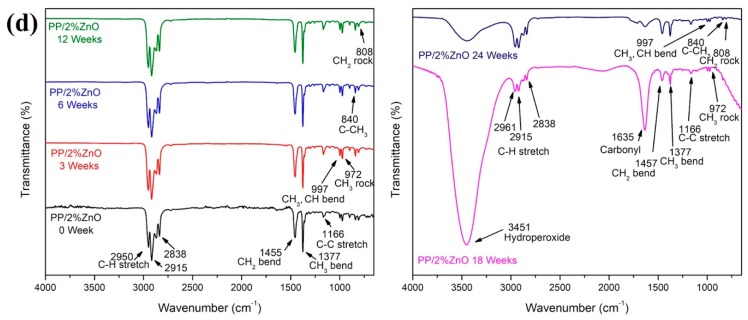
Influence of exposure to sunlight on the chemical structure of the polymer in PP/ZnO nanocomposites investigated by FTIR: (**a**) PP/0%ZnO and (**b**) PP/0.5%ZnO. (**c**) PP/1%ZnO and (**d**) PP/2%ZnO.

All PP/ZnO nanocomposites show the chemical structure of PP which consists of a linear structure of carbon atoms connected with methyl (CH_3_) groups [36] (Figure 7). Peaks of C–H, CH_3_, and C–C, at 2900, 1377, and 1166 cm^−1^, respectively, were obviously seen in the PP/0%ZnO samples at 0 weeks.

The changes of the chemical structure PP/ZnO nanocomposites under exposure to sunlight can be divided into the following two phases: In the first phase, 3–12 weeks, the chemical structure of composites (i.e., the peaks of the C–H, CH_3_, and C–C at 2900, 1377, and 1166 cm^−1^, respectively) could be observed similarly to the beginning (0 week). For the other phase, 18–24 weeks, the chemical structure of PP/ZnO nanocomposites changed significantly, i.e., peaks at 3600–3200 and 1800–1200 cm^−1^ that represent hydroperoxide and carbonyl species (such as ketone, ester, and carboxylic acid), functional groups, respectively, appeared. Significant C–H, CH_3_, and C–C peaks of PP still appeared as well.

**Figure 8:**
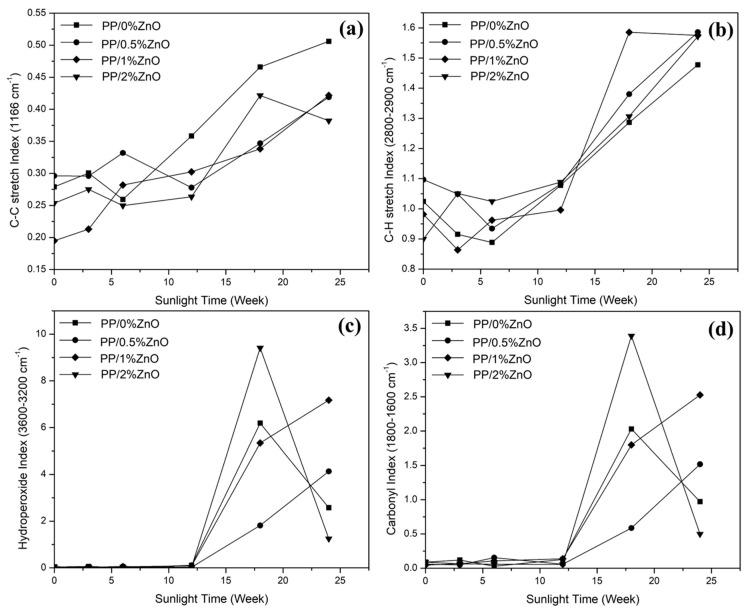
Chemical structure indexes of PP/ZnO nanocomposites during exposure to sunlight: (**a**) C–C bond, (**b**) C–H bond, (**c**) hydroperoxide, and (**d**) carbonyl group.

In order to perform a quantitative study, the chemical structure indexes of C–C bond, C-H bond, hydroperoxide, and carbonyl species were calculated using the peak at 1377 cm^−1^ (C–H bending of CH_2_) as the reference. The results are shown in Figure 8. It was found that the C–C stretching gradually increased with the exposure time, while C–H stretching was found to decrease a bit from 3 to 12 weeks and after that increased significantly during 12–24 weeks. Differently, hydroperoxide and carbonyl species exhibited the increase during 18–24 weeks. With ZnO, the composites exhibited higher hydroperoxide and carbonyl species compared with neat PP.

Based on the results of chemical structure changes, we could predicate that sunlight (the outdoor environment) has a significant influence on the degradation of PP/ZnO nanocomposites. This result can obviously be observed after 12 weeks and is consistent with the mechanical property changes discussed above. Some PP/ZnO nanocomposites show unsteady chemical structure changes (i.e., hydroperoxide and carbonyl species at and after 18 weeks), indicating that ZnO nanoparticles act as light screens to protect the PP molecules from UV [4,17]. This shows delayed changes in the chemical structure.

**Figure 9:**
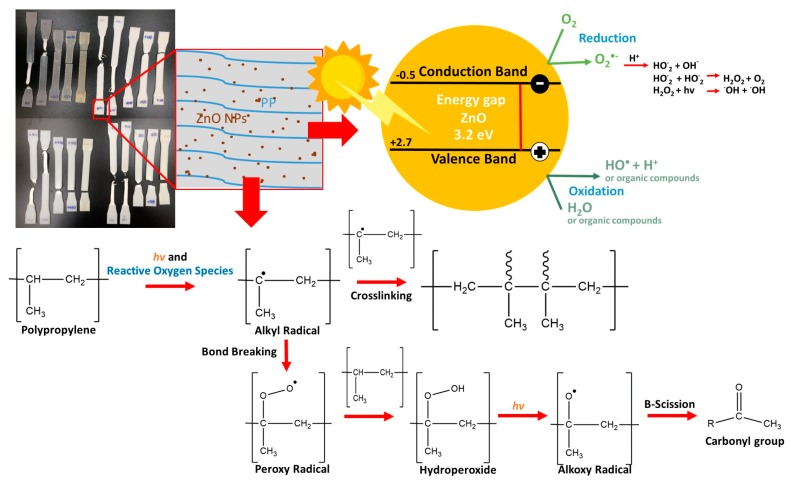
Mechanism of photocatalytic oxidations and polymer degradation in PP/ZnO nanocomposites.

ZnO in PP composites under exposure to sunlight exhibited photocatalytic degradation. Detailed reaction mechanisms which explain this phenomenon of nanocomposites in PP materials were proposed by Bustos-Torres et al. [2], Sirelkhatim et al. [21], and Gutiérrez-Villarreal and Zavala-Betancourt [37]. Figure 9 shows a proposed mechanism for photocatalytic polymer degradation with crosslink and bond breaking in PP/ZnO nanocomposites. When a photon (hν) matches or exceeds the band gap energy of ZnO (3.2 eV, from conduction band (CB) and valence band (VB) of about −0.5 eV and about 2.7 eV, respectively) [8], electrons can be activated and produce oxidative species. Alkyl radicals can be subsequently generated by the oxidative species. Crosslinking can be generated from the alkyl radicals to produce C–C bonds.

Meanwhile, bonds with alkyl radicals can also break by reacting with O_2_ to produce peroxy radicals. Peroxy radicals can form unstable hydroperoxide groups and be decomposed to produce alkoxy radicals and hydroxy radicals. Then alkoxy radicals promote scissions in the PP backbone, oxidation, and generation of carbonyl products [10]. These processes are believed to be the major causes of the changes in both the mechanical properties and chemical structures of the PP/ZnO nanocomposites observed in this work. The cross-linked molecules are the causes of: (1) the increasing mechanical properties (Young’s modulus and stress at yield point) after short time exposure to sun light observed in the tensile test results; and (2) the increase of C–C bonds seen in the FTIR spectra. On the other hand, at sunlight exposure times longer than 18 weeks, the Young’s modulus and stress at yield point were found to reduce, because of degradation of the PP matrix. This caused the significant changes in hydroperoxide and carbonyl groups in the composites inferred from the FTIR spectra.

#### 3.2.3. Photocatalytic Activity

**Figure 10:**
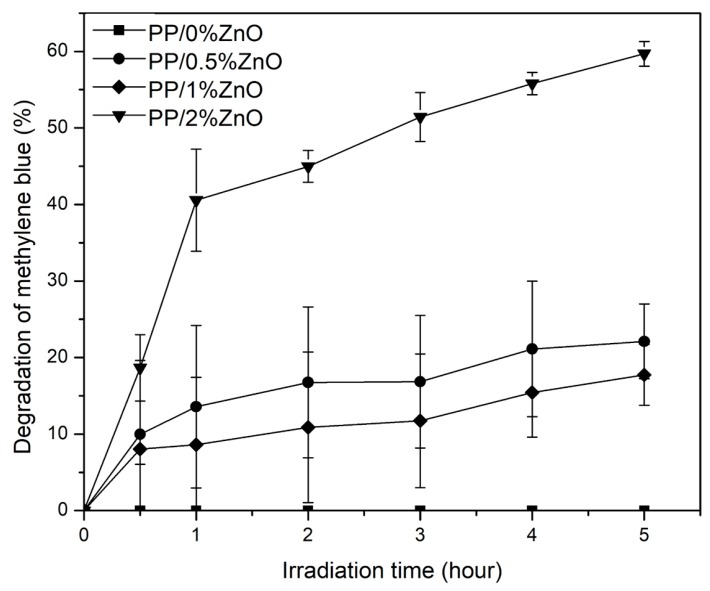
Photocatalytic degradation of MB under UVA irradiation (λ_max_ = 365 nm) of PP/ZnO nanocomposites (MB concentration = 25 mg/L, nanocomposite content = 10 g/100 mL) (note: error bars are standard deviations).

To demonstrate an application of PP/ZnO nanocomposites in the photocatalytic field, the photocatalytic degradation of methylene blue in solutions under UVA irradiation was investigated using PP/ZnO nanocomposite samples as the catalysts. Figure 10 shows the relationship between photocatalytic degradation of MB at different reaction times. The percentage of MB degradation was directly related to the irradiation time which can confirm the UV-vis absorption of nanocomposite materials [38]. PP/1%ZnO and PP/0.5%ZnO nanocomposites exhibited MB degradations of about 15%–20% within 5 h. The error bars of both nanocomposites are overlapped, so the MB degradations are not statistically different. This is due to the nature of nanocomposites with low contents of the filler. The ZnO photocatalysts could contact MB solution at only the surface of the nanocomposites, caused relatively not high photocatalytic MB degradations at low contents of ZnO. PP/2%ZnO nanocomposite exhibited the highest photocatalytic activity among all of the nanocomposites with 59% of degradation of MB within 5 h.

From the result, it can be seen that PP/ZnO nanocomposites in this work can be used in photocatalytic applications through the interfacial reaction of ZnO to oxidize the dye molecules [38]. It can imply that it may be possible to further use the nanocomposites to inhibit organisms [21,39], somect we examined the antibacterial activity. In addition, light exposure may induce photocorrosion of semiconductors, that lead to exhibit relatively low chemical stability of nanocomposites, due to the photogenerated electrons and holes that can participate in decomposing the semiconductor [40]. Photocorrosion in Ag_3_PO_4_/ZnO [41] and ZnO/PMMA [42] nanocomposites was reported. It was reported previously that photocatalytic cycle activity as an indication of photostability can be one of factors that can indirectly indicate semiconductor corrosion [40]. In this work, recycling tests of nanocomposites for photocatalytic MB degradation were performed up to 3 cycles. It was found that the MB degradation of PP/ZnO composites remained at the same efficiency (59% ± 4%, 59% ± 3%, and 58% ± 6% within 5 h, for 1–3 recycles, respectively). This could preliminary imply that ZnO has photostability in the nanocomposites.

#### 3.2.4. Antibacterial Activity

**Figure 11:**
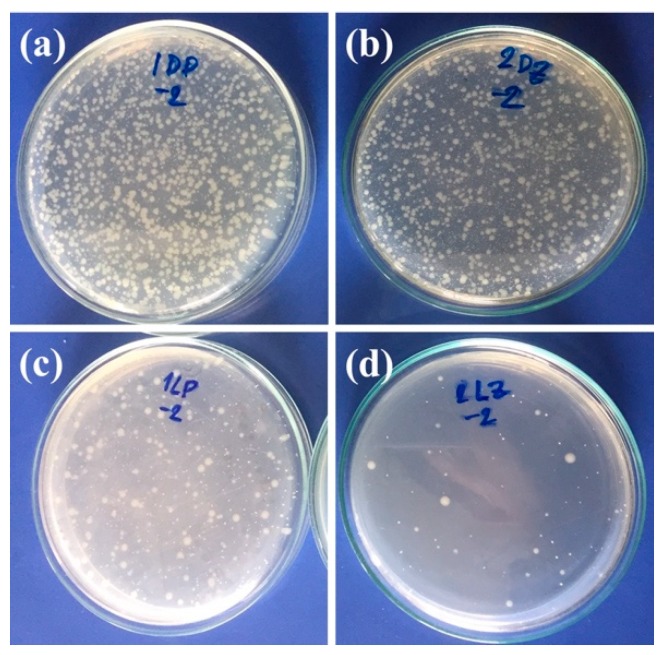
Antibacterial activity test under dark [(**a**) PP/0%ZnO and (**b**) PP/2%ZnO] and light condition [(**c**) PP/0%ZnO and (**d**) PP/2%ZnO].

**Figure 12:**
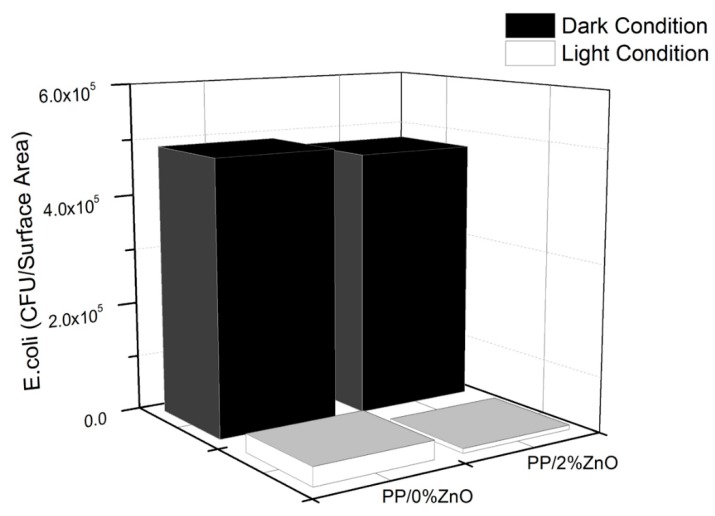
Antibacterial activity test of PP/0%ZnO and PP/2%ZnO under dark and light condition.

Reactive oxygen species, such as hydroxy radicals (HO), hydrogen peroxide (H_2_O_2_), and superoxide anion (O_2_^−^), generated during exposure of PP/ZnO nanocomposites to sunlight not only cause the changes in mechanical properties and chemical structures, they could be also successfully applied to completely destroy organic compounds turning them into CO_2_ and H_2_O and the cell membranes of bacteria [3,30,43]. To prove this, the antibacterial activity (against *E. coli*) of PP/0%ZnO (neat PP) and PP/2%ZnO was examined under dark and light conditions as shown in Figure 11 and Figure 12. PP/2%ZnO could inhibit the growth of *E. coli* compared with PP/0%ZnO, as indicated by 4.8 × 10^5^ and 8.2 × 10^3^ CFU/surface area results under dark and light conditions, respectively (compared with the 4.9 × 10^5^ and 3.4 × 10^4^ CFU/surface area, respectively, of the PP/0%ZnO case).

Generally, it has been observed that the antibacterial activity of ZnO to inhibit bacterial growth could occur under light conditions as well as dark conditions [21]. In terms of dark conditions, the antibacterial activity of PP/2%ZnO in the dark was caused because of the cell walls of *E. coli* are in direct contact with ZnO [21] and the release f Zn ions (Zn^2+^) can effectively destroy the cell membrane activity of the *E. coli* [43]. Besides, under light conditions, Zn^2+^ also react with oxygen (O_2_) in the air and water leading to the generation of reactive oxygen species and HO^.^ which destroy the bacterial cell membranes [30]. Besides, under light conditions, Zn^2+^ also reacted with O_2_ in the air and H_2_O leading to reactive oxygen species (HO, H_2_O_2_, O_2_^−^) through a photocatalytic activity that destroys the bacteria cell membranes [30].

## 4. Conclusions

Nanocomposite materials were prepared by compounding PP with ZnO nanoparticles. Dumbbell-shaped specimens of the composites were prepared. The influence of ZnO nanoparticle content on the morphology, mechanical properties, chemical structure, photocatalytic activity, and antibacterial properties of the obtained nanocomposites was investigated. SEM images showed that the ZnO nanoparticles were well distributed in the PP matrix. Characterizations of the mechanical properties and chemical structures before and after sunlight exposure found that at the shortest exposure time, crosslinks could occur in the nanocomposites, which resulted in improved mechanical properties. However, sunlight exposure for time periods longer than 18 weeks caused a reduction of the mechanical properties, due to degradation of the PP matrix. It was found that PP with 2% ZnO could achieved the photocatalytic degradation of methylene blue, a model organic dye, by up to 59%. The result of photocatalytic antibacterial tests indicated that the PP/ZnO nanocomposites had better antibacterial properties than neat PP. This result will be very beneficial for the development of nanocomposite materials and solving the problem of plastics in environments that can be applied in many photocatalytic applications, e.g., containers for the degradation of wastewater, etc.
